# Supplementary material for: Bacterial IF2’s N-terminal IDR drives cold-induced phase separation and promotes fitness during cold stress
Source: bioRxiv. 2025 Feb 2:2025.02.02.631968. Preprint. [Version 1] doi: 10.1101/2025.02.02.631968 (PMC11838494; doi:10.1101/2025.02.02.631968)
Supplement: Supplement 1 [file media-1.pdf]

Supplementary Figure Legends

Fig S1.
Bar graphs show turbidity increases with increasing IF-2 concentration, reaching a plateau at the basal IF-2 concentration (20 μM) in the absence of RNA. In the presence of varying RNA concentrations, turbidity initially increases but decreases at higher RNA concentrations, indicating a concentration-dependent modulation of phase separation by RNA.

Fig S2.
In the presence of 5 μM IF-2 and 5 ng/μL total RNA, an increase in turbidity was observed. However, the addition of excess KCl or RNase A resulted in a decrease in turbidity, indicating that the turbidity induced by the IF-2 protein is reversible under these conditions.

Fig S3.
20 μM IF-2 tagged MBP protein was incubated in a buffer containing 20 mM Tris (pH 7.4), 75 mM KCl, 10 mM $MgCl_2$, and 1 mM DTT for 2.5 hours at room temperature. Under these conditions, the protein did not undergo phase separation. However, upon the addition of TEV protease at a 1:15 molar ratio and incubation for 2.5 hours, the IF-2 protein exhibited clear phase separation. This suggests that the cleavage of the MBP tag by TEV protease promotes the phase separation of IF-2.

Fig S4.
125 μM MANT-GTP was incubated with 1 μM IF2, 1 μM IF2ΔNTD, 1 μM IF2β, or 1 μM Aconitase and measured in a fluorescence plate reader. Fluorescence enhancement at 450 nm was calculated as a ratio of the MANT-GTP fluorescence in the presence of each protein relative relative to the MANT-GTP fluorescence intensity. As a control, proteins samples were also incubated with 3mM GTP as an unlabeled chase. Protein samples lacking MANT-GTP were run as negative controls. Error bars represent standard deviation from three independent replicate measurements. * indicates $p<0.05$ from 1 sided t-test with uneven variance.

Fig S5.
Purified 5 μM IF-2 β isoforms was incubated with 5 ng/μL total RNA in a buffer containing 20 mM Tris (pH 7.4), 75 mM KCl, 10 mM MgCl2, and 1 mM DTT at room temperature. The bar graph represents the percentage of image area covered by IF-2 RNA droplets, quantified using ImageJ. Values shown are the averages of three independent replicates, each consisting of >50 droplets. Statistically significant differences between full length and β isoforms were determined using a one-tailed t-test for samples with unequal variance.

Fig S6.
Purified 5 μM IF-2 was incubated with 5 ng/μL total RNA and 1 mM GTP or GDP in a buffer containing 20 mM Tris (pH 7.4), 75 mM KCl, 10 mM MgCl2, and 1 mM DTT at room temperature. The presence of nucleotides led to a reduction in droplet formation or the appearance of aggregates. The bar graph represents the percentage of image area covered by IF-2 RNA droplets, quantified using ImageJ. Values shown are the averages of three independent replicates, each consisting of >50 droplets. Statistically significant differences between conditions with and without nucleotides were determined using a one-tailed t-test for samples with unequal variance.

Fig S7.
Comparison of the growth assay between the infB-SPA strain and the wild-type MG1655 strain. Cells were grown to log phase (OD_{600} = 0.5), serially diluted in a 1:10 ratio, and spotted onto

plates. The plates were incubated overnight at 37°C. No growth difference was observed between the tagged infB-SPA strain and the MG1655 wild-type strain. The liquid growth curve assay also shows that there is no significant difference between them and doubling time of infB-SPA strain is 37.8 minutes ± 3.3 minutes and MG1655 wild-type is 33.4 minutes ± 1.8 minutes (2-sided t-test of unequal variance P-value is 0.14). Error bars represent the standard deviation in doubling times.

Fig S8. Immunofluorescence analysis was performed on wild-type MG1655 to check the specificity of the primary antibody. Since the wild-type MG1655 lacks the SPA-tagged IF-2, no fluorescence signal was detected in these cells, in contrast to the infB-SPA tagged strain, which showed a clear signal. This confirms the specificity of the primary antibody for the SPA-tagged IF-2.

Fig S9. Western blot analysis of infB-SPA and BL21 strains using a primary antibody to confirm the conjugation of the SPA tag with the infB gene. The molecular weight of IF-2 is 97 kDa, but due to the presence of the SPA tag, the observed band corresponds to approximately 130 kDa. No band is detected in the BL21 strain, which lacks the SPA tag.

Fig S10. List of psychrophilic and thermophilic bacterial species and references for their characterization.

Fig S1

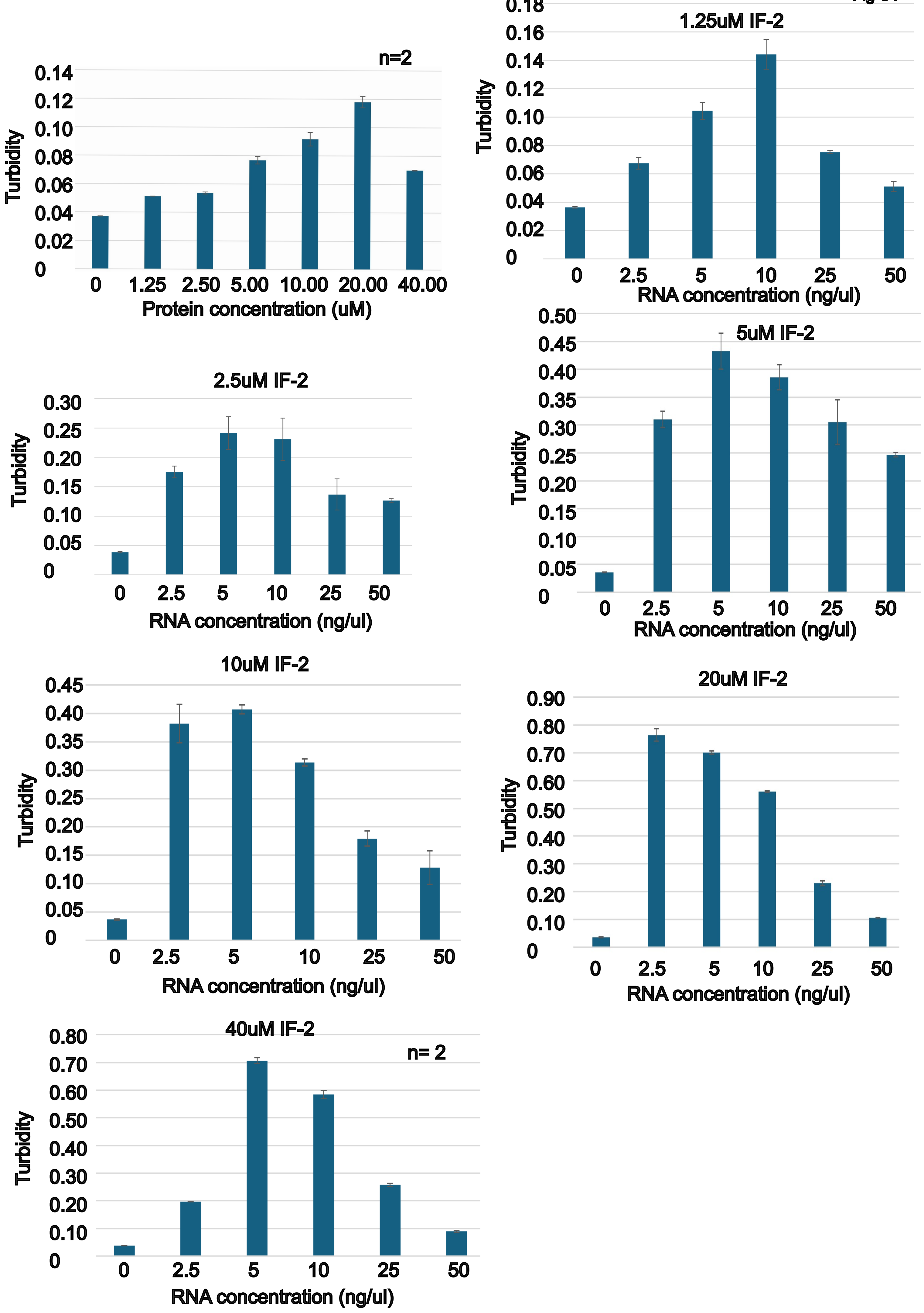
n=2
Turbidity
0.14
0.12
0.10
0.08
0.06
0.04
0.02
0
0
1.25
2.50
5.00
10.00
20.00
40.00
Protein concentration (uM)
1.25uM IF-2
0.18
0.16
0.14
0.12
0.10
0.08
0.06
0.04
0.02
0
2.5
5
10
25
50
RNA concentration (ng/ul)
2.5uM IF-2
0.30
0.25
0.20
0.15
0.10
0.05
5uM IF-2
0.50
0.45
0.40
0.35
10uM IF-2
20uM IF-2
0.90
0.80
0.70
0.60
0.50
0.40
0.30
0.20
0.10
40uM IF-2
n= 2

Fig S2

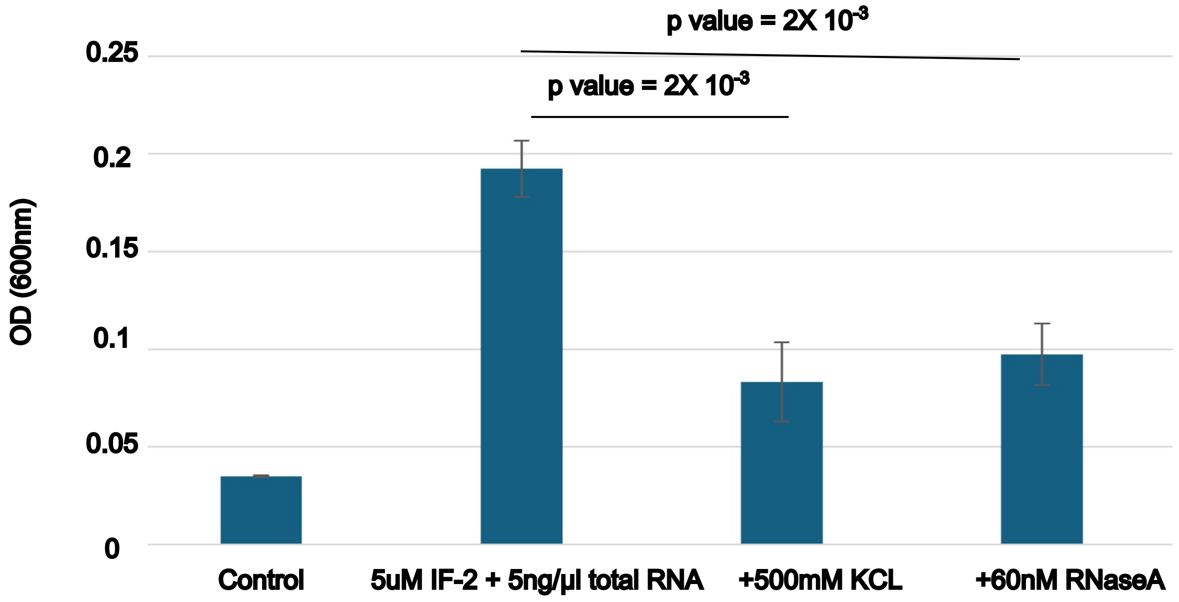
p value = 2X 10-3
p value = 2X 10-3
0.25
0.2
0.15
0.1
0.05
0
OD (600nm)
Control
5uM IF-2 + 5ng/µl total RNA
+500mM KCL
+60nM RNaseA

Fig S3

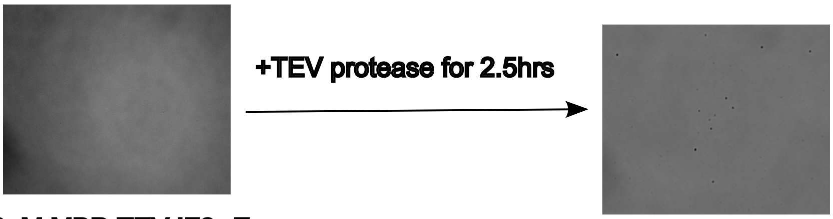

20uM MBP-TEV-IF2_*Ec*

Fig S4

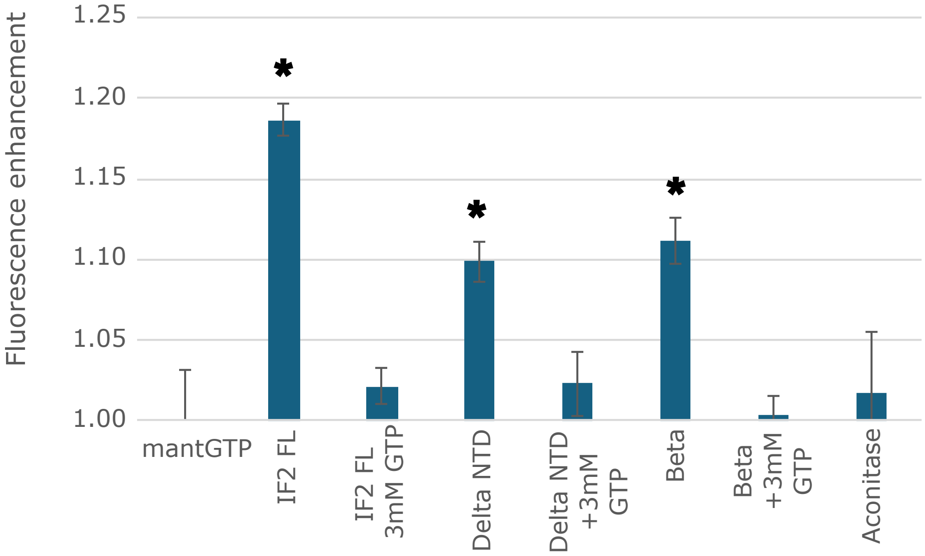

Fluorescence enhancement
1.25
1.20
1.15
1.10
1.05
1.00
*
*
*
mantGTP
IF2 FL
IF2 FL 3mM GTP
Delta NTD
Delta NTD +3mM GTP
Beta
Beta +3mM GTP
Aconitase

Fig S5

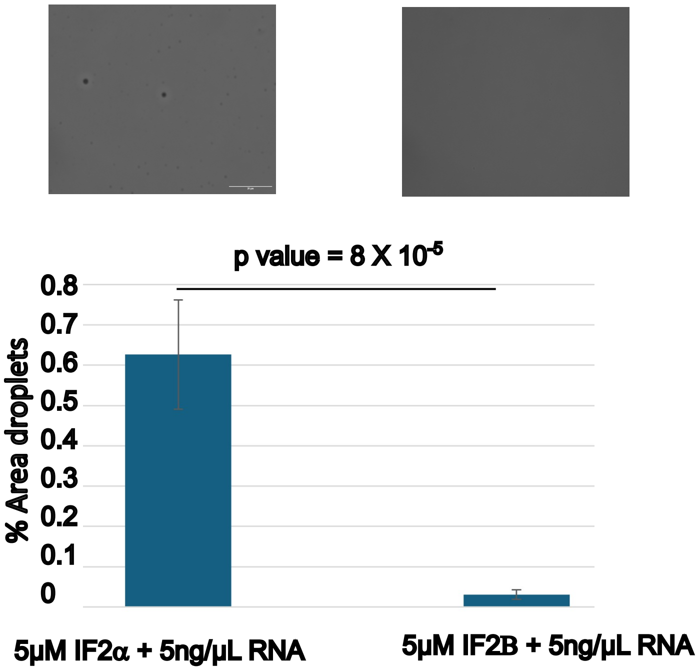
p value = 8 X 10-5
% Area droplets
0.8
0.7
0.6
0.5
0.4
0.3
0.2
0.1
0
5μM IF2α + 5ng/μL RNA
5μM IF2β + 5ng/μL RNA

Fig S6

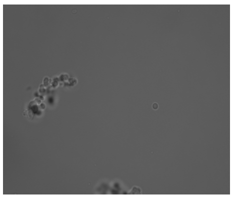

5µM IF-2 + 5ng/µL RNA +1mM GTP

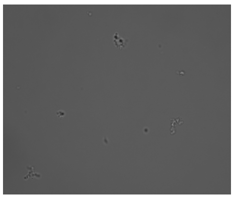

5µM IF-2 + 5ng/µL RNA + 1mM GDP

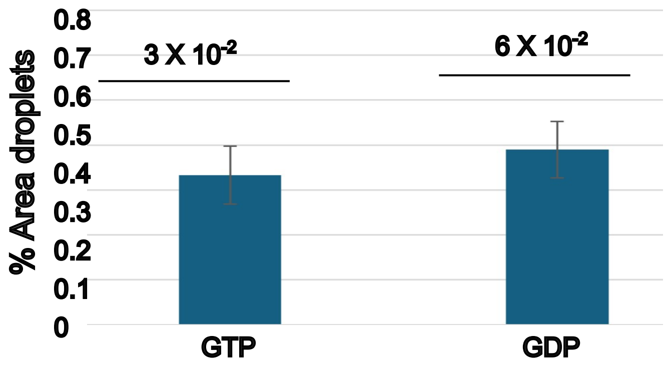

Fig S7

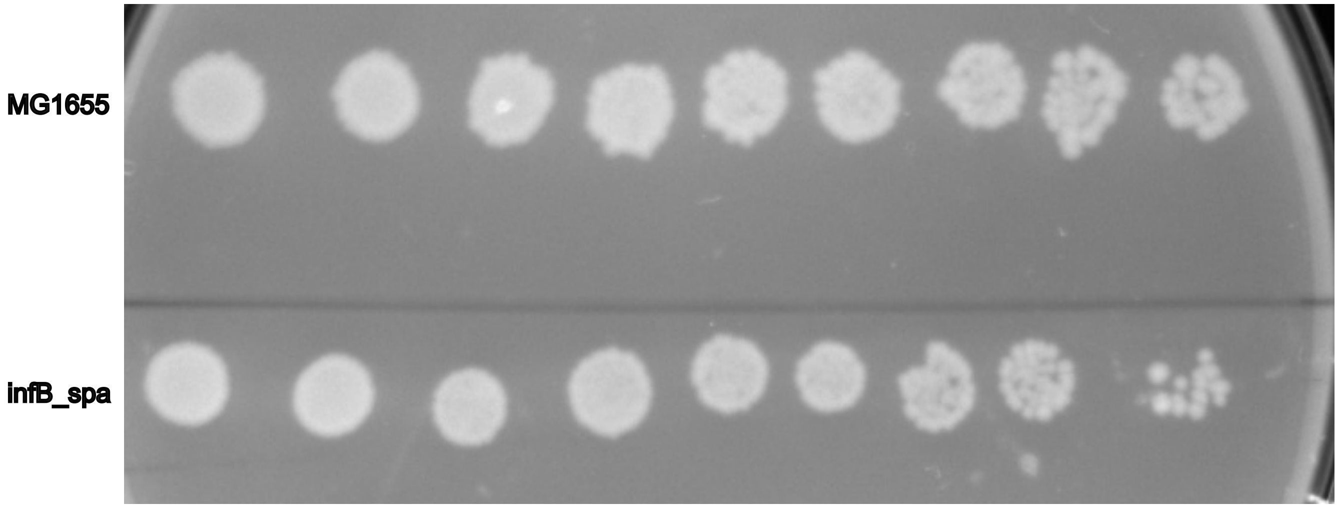

MG1655
infB_spa

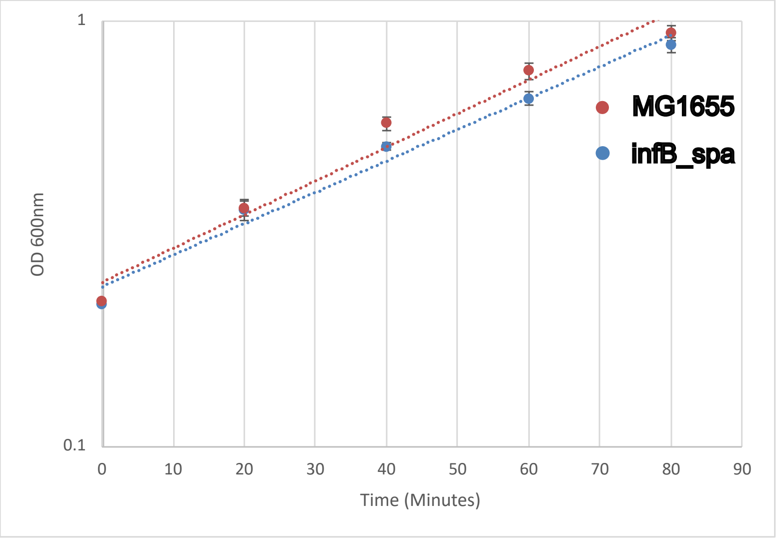

1
0.1
OD 600nm
0
10
20
30
40
50
60
70
80
90
Time (Minutes)
MG1655
infB_spa

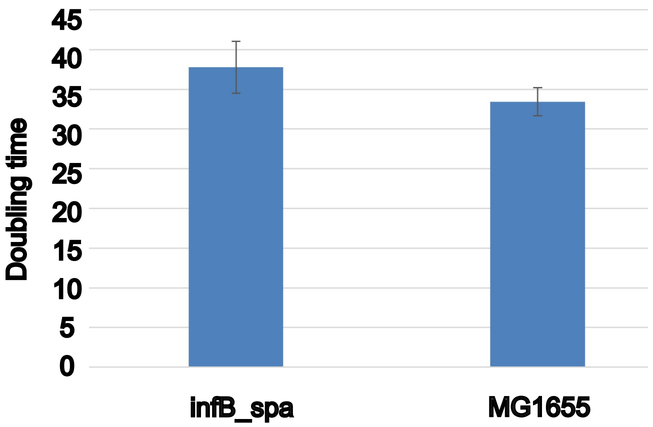

45
40
35
30
25
20
15
10
5
0
Doubling time
infB_spa
MG1655

Fig S8

mouse-anti-FLAG primary
anti-mouse-CY5 secondary

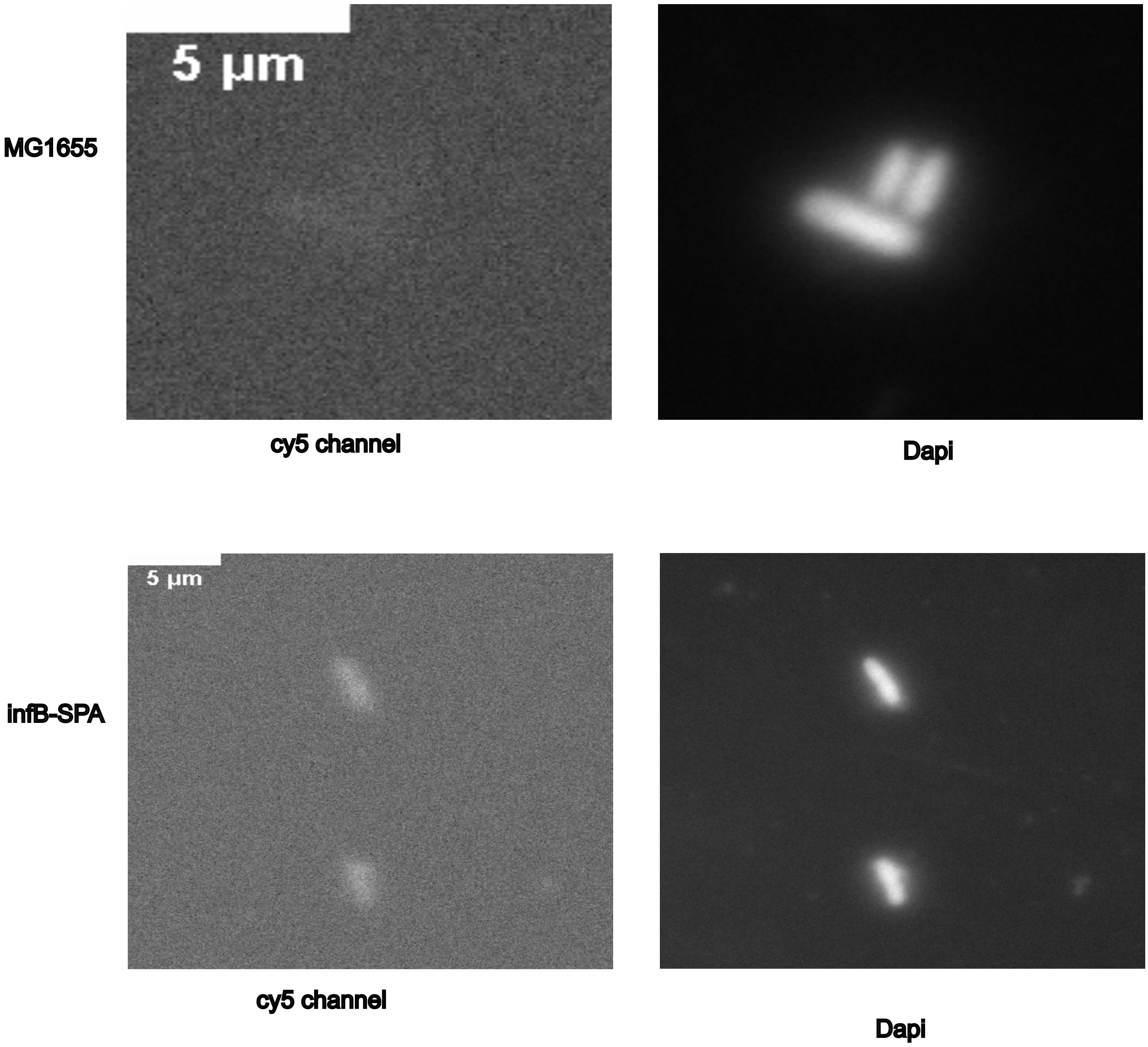

Fig S9

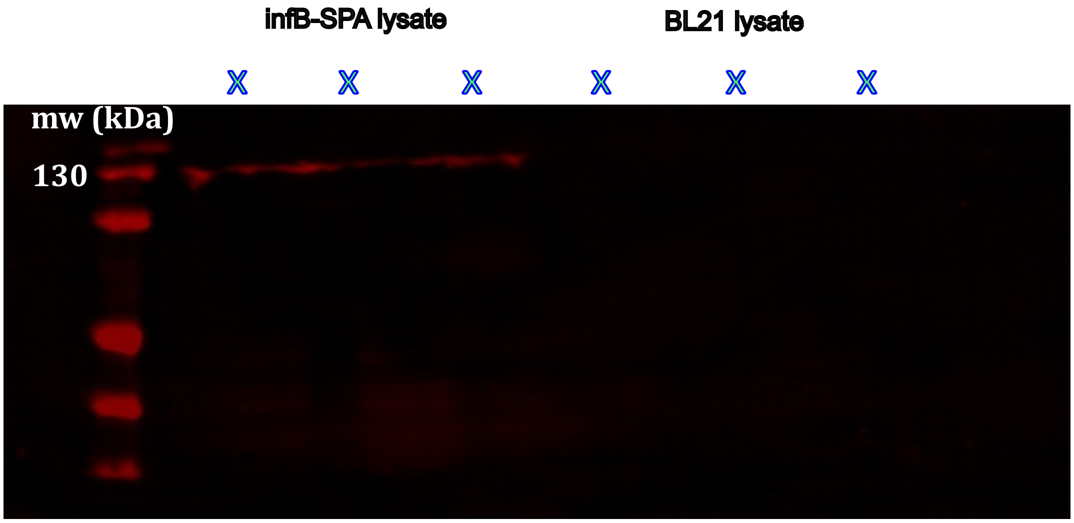

Name	Type	Reference
Pseudorhodobacter antarcticus	Psychrophile	[1]
Shewanella livingstonensis	Psychrophile	[2]
Psychrobacter cryohalolentis K5	Psychrophile	[3, 4]
Colwellia psychrerythraea 34H	Psychrophile	[3, 5]
Psychromonas ingrahamii 37	Psychrophile	[6]
Psychrobacter arcticus 273-4	Psychrophile	[7, 8]
Flavobacterium psychrophilum JIP02/86	Psychrophile	[9]
Desulfotalea psychrophila LSv54	Psychrophile	[10, 11]
Pseudoalteromonas atlantica T6c	Psychrophile	[11]
Renibacterium salmoninarum ATCC 33209	Psychrophile	[11]
Octadecabacter arcticus	Psychrophile	[12]
Octadecabacter antarcticus	Psychrophile	[12]
Thermus thermophilus HB27	Thermophile	[13]
Caldicellulosiruptor saccharolyticus DSM 8903	Thermophile	[14]
Thermus scotoductus SA-01	Thermophile	[15]
Fervidobacterium nodosum Rt17-B1	Thermophile	[16]
Thermodesulfobacterium commune DSM 2178	Thermophile	[17]
Hydrogenobacter thermophilus TK-6	Thermophile	[18]
Thermotoga maritima MSB8	Thermophile	[19]
Elioraea tepidiphila	Thermophile	[20]
Tepidamorphus gemmatus	Thermophile	[21]
Rubellimicrobium thermophilum	Thermophile	[22]

(1) Chen, C. X.; Zhang, X. Y.; Liu, C.; Yu, Y.; Liu, A.; Li, G. W.; Li, H.; Chen, X. L.; Chen, B.; Zhou, B. C.; et al. Pseudorhodobacter antarcticus sp. nov., isolated from Antarctic intertidal sandy sediment, and emended description of the genus Pseudorhodobacter Uchino et al. 2002 emend. Jung et al. 2012. *Int J Syst Evol Microbiol* **2013**, *63* (Pt 3), 849-854. DOI: 10.1099/ijs.0.042184-0 From NLM.

(2) Luo, G.; Fujii, S.; Koda, T.; Tajima, T.; Sambongi, Y.; Hida, A.; Kato, J. Unexpectedly high thermostability of an NADP-dependent malic enzyme from a psychrophilic bacterium, Shewanella livingstonensis Ac10. *J Biosci Bioeng* **2021**, *132* (5), 445-450. DOI: 10.1016/j.jbiosc.2021.07.005 From NLM.

(3) Amato, P.; Christner, B. C. Energy metabolism response to low-temperature and frozen conditions in Psychrobacter cryohalolentis. *Appl Environ Microbiol* **2009**, *75* (3), 711-718. DOI: 10.1128/aem.02193-08 From NLM.

(4) Smith, D. J.; Schuerger, A. C.; Davidson, M. M.; Pacala, S. W.; Bakermans, C.; Onstott, T. C. Survivability of Psychrobacter cryohalolentis K5 under simulated martian surface conditions. *Astrobiology* **2009**, *9* (2), 221-228. DOI: 10.1089/ast.2007.0231 From NLM.

(5) Czajka, J. J.; Abernathy, M. H.; Benites, V. T.; Baidoo, E. E. K.; Deming, J. W.; Tang, Y. J. Model metabolic strategy for heterotrophic bacteria in the cold ocean based on Colwellia psychrerythraea 34H. *Proc Natl Acad Sci U S A* **2018**, *115* (49), 12507-12512. DOI: 10.1073/pnas.1807804115 From NLM.

(6) Auman, A. J.; Breezee, J. L.; Gosink, J. J.; Kämpfer, P.; Staley, J. T. Psychromonas ingrahamii sp. nov., a novel gas vacuolate, psychrophilic bacterium isolated from Arctic polar sea ice. *Int J Syst Evol Microbiol* **2006**, *56* (Pt 5), 1001-1007. DOI: 10.1099/ijs.0.64068-0 From NLM.

(7) Ayala-del-Río, H. L.; Chain, P. S.; Grzymski, J. J.; Ponder, M. A.; Ivanova, N.; Bergholz, P. W.; Di Bartolo, G.; Hauser, L.; Land, M.; Bakermans, C.; et al. The genome sequence of Psychrobacter arcticus 273-4, a psychroactive Siberian permafrost bacterium, reveals mechanisms for adaptation to low-temperature growth. *Appl Environ Microbiol* **2010**, *76* (7), 2304-2312. DOI: 10.1128/aem.02101-09 From NLM.

(8) Casillo, A.; Ziaco, M.; Lindner, B.; Parrilli, E.; Schwudke, D.; Holgado, A.; Beyaert, R.; Lanzetta, R.; Tutino, M. L.; Corsaro, M. M. Lipid A structural characterization from the LPS of the Siberian psychro-tolerant Psychrobacter arcticus 273-4 grown at low temperature. *Extremophiles* **2018**, *22* (6), 955-963. DOI: 10.1007/s00792-018-1051-6 From NLM.

(9) Hesami, S.; Metcalf, D. S.; Lumsden, J. S.; Macinnes, J. I. Identification of cold-temperature-regulated genes in Flavobacterium psychrophilum. *Appl Environ Microbiol* **2011**, *77* (5), 1593-1600. DOI: 10.1128/aem.01717-10 From NLM.

(10) Rabus, R.; Ruepp, A.; Frickey, T.; Rattei, T.; Fartmann, B.; Stark, M.; Bauer, M.; Zibat, A.; Lombardot, T.; Becker, I.; et al. The genome of Desulfotalea psychrophila, a sulfate-reducing bacterium from permanently cold Arctic sediments. *Environ Microbiol* **2004**, *6* (9), 887-902. DOI: 10.1111/j.1462-2920.2004.00665.x From NLM.

(11) Metpally, R. P. R.; Reddy, B. V. B. Comparative proteome analysis of psychrophilic versus mesophilic bacterial species: Insights into the molecular basis of cold adaptation of proteins. *BMC Genomics* **2009**, *10* (1), 11. DOI: 10.1186/1471-2164-10-11.

(12) Vollmers, J.; Voget, S.; Dietrich, S.; Gollnow, K.; Smits, M.; Meyer, K.; Brinkhoff, T.; Simon, M.; Daniel, R. Poles apart: Arctic and Antarctic Octadecabacter strains share high genome plasticity and a new type of xanthorhodopsin. *PLoS One* **2013**, *8* (5), e63422. DOI: 10.1371/journal.pone.0063422 From NLM.

(13) Kirchner, L.; Müller, V.; Averhoff, B. A temperature dependent pilin promoter for production of thermostable enzymes in Thermus thermophilus. *Microb Cell Fact* **2023**, *22* (1), 187. DOI: 10.1186/s12934-023-02192-1 From NLM.

(14) Talluri, S.; Raj, S. M.; Christopher, L. P. Consolidated bioprocessing of untreated switchgrass to hydrogen by the extreme thermophile Caldicellulosiruptor saccharolyticus DSM 8903. *Bioresour Technol* **2013**, *139*, 272-279. DOI: 10.1016/j.biortech.2013.04.005 From NLM.

(15) Cockrell, A. L.; Fitzgerald, L. A.; Cusick, K. D.; Barlow, D. E.; Tsoi, S. D.; Soto, C. M.; Baldwin, J. W.; Dale, J. R.; Morris, R. E.; Little, B. J.; et al. Differences in Physical and Biochemical Properties of Thermus scotoductus SA-01 Cultured with Dielectric or Convection Heating. *Appl Environ Microbiol* **2015**, *81* (18), 6285-6293. DOI: 10.1128/aem.01618-15 From NLM.

(16) Yang, Y.; Zhu, Y.; Obaroakpo, J. U.; Zhang, S.; Lu, J.; Yang, L.; Ni, D.; Pang, X.; Lv, J. Identification of a novel type I pullulanase from Fervidobacterium nodosum Rt17-B1, with high thermostability and suitable optimal pH. *Int J Biol Macromol* **2020**, *143*, 424-433. DOI: 10.1016/j.ijbiomac.2019.10.112 From NLM.

(17) Bhatnagar, S.; Badger, J. H.; Madupu, R.; Khouri, H. M.; O'Connor, E. M.; Robb, F. T.; Ward, N. L.; Eisen, J. A. Genome Sequence of a Sulfate-Reducing Thermophilic Bacterium, Thermodesulfobacterium commune DSM 2178T (Phylum Thermodesulfobacteria). *Genome Announc* **2015**, *3* (1). DOI: 10.1128/genomeA.01490-14 From NLM.

(18) Ishii, M.; Igarashi, Y.; Kodama, T. Purification and characterization of ATP:citrate lyase from Hydrogenobacter thermophilus TK-6. *J Bacteriol* **1989**, *171* (4), 1788-1792. DOI: 10.1128/jb.171.4.1788-1792.1989 From NLM.

(19) Wang, Z.; Tong, W.; Wang, Q.; Bai, X.; Chen, Z.; Zhao, J.; Xu, N.; Liu, S. The temperature dependent proteomic analysis of Thermotoga maritima. *PLoS One* **2012**, *7* (10), e46463. DOI: 10.1371/journal.pone.0046463 From NLM.

(20) Saini, M. K.; Yoshida, S.; Sebastian, A.; Hara, E.; Tamaki, H.; Soulier, N. T.; Albert, I.; Hanada, S.; Tank, M.; Bryant, D. A. Elioraea tepida, sp. nov., a Moderately Thermophilic Aerobic Anoxygenic Phototrophic Bacterium Isolated from the Mat Community of an Alkaline Siliceous Hot Spring in Yellowstone National Park, WY, USA. *Microorganisms* **2022**, *10* (1), 80.

(21) Albuquerque, L.; Rainey, F. A.; Pena, A.; Tiago, I.; Veríssimo, A.; Nobre, M. F.; da Costa, M. S. Tepidamorphus gemmatus gen. nov., sp. nov., a slightly thermophilic member of the Alphaproteobacteria. *Syst Appl Microbiol* **2010**, *33* (2), 60-66. DOI: 10.1016/j.syapm.2010.01.002 From NLM.

(22) Denner, E. B. M.; Kolari, M.; Hoornstra, D.; Tsitko, I.; Kämpfer, P.; Busse, H. J.; Salkinoja-Salonen, M. Rubellimicrobium thermophilum gen. nov., sp. nov., a red-pigmented, moderately thermophilic bacterium isolated from coloured slime deposits in paper machines. *Int J Syst Evol Microbiol* **2006**, *56* (Pt 6), 1355-1362. DOI: 10.1099/ijs.0.63751-0 From NLM.

(23) Altschul, S. F.; Gish, W.; Miller, W.; Myers, E. W.; Lipman, D. J. Basic local alignment search tool. *J Mol Biol* **1990**, *215* (3), 403-410. DOI: 10.1016/s0022-2836(05)80360-2 From NLM.

(24) Sayers, E. W.; Bolton, E. E.; Brister, J. R.; Canese, K.; Chan, J.; Comeau, D. C.; Connor, R.; Funk, K.; Kelly, C.; Kim, S.; et al. Database resources of the national center for biotechnology information. *Nucleic Acids Res* **2022**, *50* (D1), D20-d26. DOI: 10.1093/nar/gkab1112 From NLM.

(25) Emenecker, R. J.; Griffith, D.; Holehouse, A. S. Metapredict: a fast, accurate, and easy-to-use predictor of consensus disorder and structure. *Biophysical Journal* **2021**, *120* (20), 4312-4319. DOI: https://doi.org/10.1016/j.bpj.2021.08.039.

(26) Gasteiger E., H. C., Gattiker A., Duvaud S., Wilkins M.R., Appel R.D., Bairoch A. Protein Identification and Analysis Tools on the Expasy Server. Walker, J. M. Ed.; The Proteomics Protocols Handbook, Humana Press, 2005; pp 571-607.
